# *No one is safe! But who’s more susceptible?* Locus of control moderates pandemic perceptions’ effects on job insecurity and psychosocial factors amongst MENA hospitality frontliners: a PLS-SEM approach

**DOI:** 10.1186/s12889-021-12071-2

**Published:** 2021-11-06

**Authors:** Ali B. Mahmoud, William D. Reisel, Dieu Hack-Polay, Leonora Fuxman

**Affiliations:** 1 St. John’s University, New York, USA; 2 University of Wales Trinity Saint David, London, UK; 3 Crandall University, Moncton, New Brunswick Canada; 4 University of Lincoln, Lincoln, UK

**Keywords:** COVID-19, Personality, Individual differences, Locus of control, Job insecurity, Psychosocial factors, Hospitality sector, Middle East, North Africa

## Abstract

**Background:**

The research aimed to formulate and test a model concerning COVID-19 perceptions effects on job insecurity and a set of psychosocial factors comprising anxiety, depression, job burnout and job alienation in the Middle East and North African (hereafter, MENA) regional context. Also, the study attempted to examine whether locus of control can moderate these hypothesised linkages amongst customer service employees working in MENA hospitality organisations.

**Methods:**

The study is based on a sample of 885 responses to an online survey and Partial Least Square Structural Equation Modelling (PLS-SEM).

**Results:**

The main findings show the existence of a significant correlation between COVID perceptions and job insecurity and all psychosocial factors, i.e., more intense COVID-19 perceptions accompany higher levels of job insecurity, anxiety, depression, job burnout and job alienation. Furthermore, our results revealed that, in pandemic time, hospitality customer service employees with external locus of control are more likely to suffer higher alienation, anxiety and depression than those with internal locus of control.

**Conclusions:**

The research originality centres on the establishment that COVID-19 has a severe negative impact within the hospitality customer service labour force (in the MENA region). These effects were more profound for participants who claimed external locus of control than those with internal locus of control.

## Background

The Coronavirus ‘COVID-19’ has provoked anxiety in society. This has been particularly severe for the hospitality sector, which came near to collapse, especially in developing and emerging economies [1]. Whilst investment in much of the Middle East and North Africa (MENA) region has been strained owing to regional geopolitical conflicts for many years, the region is now facing additional economic challenges due to the pandemic [2]. The sector most directly impacted has been the hospitality industry [3].

The COVID-19 era has rendered service delivery difficult or impossible in the face of social distancing requirements [4]. Businesses such as air travel, hotels and catering, etc. were driven to design dramatic measures at great costs (e.g., using artificial intelligence to sanitise their premises). Survival required compliance with government and international regulations, which sought assurances that businesses provide a hygienic and safe environment [5]. Complying with these additional safety measures has affected efforts to balance the use of machines and human employees, an ongoing debate in social sciences and business & management [6]. The robot vs human conflict has intensified because of the need for compliance and worker safety due to COVID-19 [6].

Consequently, the human workforce in the hospitality sector has been depleted, with over 40% job losses [7]. Artificial intelligence and robotics are increasingly strategically significant [8] to the detriment of traditional people resources [6]. This replacement of human labour with technological alternatives threatens mental well-being, ranging from stress to anxiety and depression) owing to the prospect of job insecurity [9].

Specifically, workplace redundancies and fear that the pandemic causes are contributing to increased job insecurity as well as psychological distress [10]. That potentially predicts decreased customer satisfaction levels [9, 11]. Therefore, this study responds to the need for additional research in workplace well-being and presents a novel model of the potential adverse correlates of COVID-19 perceptions on job insecurity anxiety, burnout, depression, and alienation of customer service personnel in MENA’s hospitality sector. According to AB Mahmoud, D Hack-Polay, L Fuxman and M Nicoletti [12], COVID-19 perceptions (or pandemic perceptions) refer to the perceived probability of discomfort and/or worry, during the COVID-19 pandemic, concerning the pandemic adverse health, economic and social ramifications articulated as disruptions to people’s pre-pandemic everyday life— describing an era characterised as the *new normal*. These issues are importance given the already precarious jobs in hospitality and tourism [13].

Since an individual’s perceived control over what is important in their life and whether the events of life are reliant upon their own actions, termed as locus of control [14, 15], this can cause variations in the way people perceive stressful conditions, locus of control is presented by previous research as a moderator of how psychosocial factors and job insecurity respond to occasions of stress [13, 16]. Individuals’ locus of control type, i.e., internal vs external, is therefore predicted to moderate the effect of COVID-19 perception. Therefore, our research also examines how a worker’s locus of control is likely to moderate such relations as we anticipate.

The research’s geographic context is significant owing to the economic and strategic significance of the tourism and hospitality sectors in the MENA region. Hospitality ranks highly in MENA behind the oil sector [17]. Moreover, the pandemic has severely reduced the tourism and hospitality industry’s viability [18]. Nevertheless, few scholarly investigations offer theoretical and empirical guidance to this threatened industry. A goal of the research is to offer MENA region hospitality businesses insights and suggestions to adapt to management practices to deal with its own COVID-19 crisis with regards to human resources.

In this paper, we first review the theoretical foundation that addresses how hospitality workers are impacted by employment and psychosocial factors, including the precarity of jobs and working conditions. Then, we explain the origins of the coronavirus pandemic and formulate our propositions. Second, we present the methods, with data collection details, participants, sample and the statistical model and analysis. Third, we present and discuss the findings before summarising the research implications, limitations, and future directions.

## Theoretical foundations

There is hardly any industry sector unaffected by the global COVID-19 pandemic. The hospitality industry has seen one of the most adverse effects of any industry sector due to its dependence on tourism and travel, which stopped suddenly throughout the world once COVID-19 gained a pandemic status. While the hospitality industry is known to be highly susceptible to many different types of crises, including catastrophic events, natural disasters, and pandemics, many sectors of the industry, for example, tourism and travel, in particular, tend to recover relatively quickly without lingering long-term decline [19, 20]. Some researchers see the current pandemic’s response to hospitality workers as consistent with the industry’s normal challenges [18]. Hospitality industry jobs are mostly high-contact jobs and, as such, are exposed to the highest risk during the pandemic and upon re-entering post-COVID [18]. While the hospitality industry is efficient with adjusting workforce levels on an as-needed basis, the unprecedented global nature and the scale of the ongoing COVID-19 crisis are expected to exacerbate job loss and subsequent insecurities in the workplace [21]. For the hospitality industry, in particular, the availability of a COVID-19 vaccine suggests that organisations will have to maintain their contingency measures at least into the intermediate future beyond the end of 2020 in an effort to minimise risk to front line employees [22].

Our interest in studying COVID-19 perceptions among hospitality industry workers was fuelled by the essential nature of many hospitality industry jobs, where employees cannot shift to the safety of remote service delivery, as is the case for back-office employees. In addition, some limited early projections suggest that organisational response to the post-COVID recovery in hotel services may shift towards innovative use of artificial intelligence and robotics [20] to replace weak human interaction during pandemic times. However, while futuristic technology-enabled solutions are expected to enhance the landscape of service jobs in many hospitality industry sectors, they are not going to replace traditional interactive service jobs. Thus, our inquiry here is designed to expand our understanding of how COVID-19 perceptions of hospitality industry employees may be associated with job insecurity and various individual psychosocial reactions.

### COVID-19 perceptions and job insecurity

Employee perception of job insecurity can result from general volatility of employment in the industry despite some organisational attempts to stabilise the rapid turnover issue by considering agility as a possible remedy [23]. For example, terms and conditions of services, as well as compensation, have been less desirable in hospitality than in many other sectors [24]. It is also well documented that work flexibility is rarely available to hospitality workers in the MENA region, with excessively burdening working hours, causing severe psychological and family issues [25, 26]. Moreover, due to the lack of alternative employment in some MENA countries and the weakness of trade unions, employees see as inevitable their poor working conditions and absence of viable options [27]. This is compounded by weak institutional frameworks that could guarantee enforceable minimum standards.

COVID-19 has caused the hospitality sector to shed millions of positions across the globe [28]. Moreover, the pandemic has exacerbated already poor job security perceptions in this industry as it has created increased fear of redundancies among service personnel [29]. We, thus, hypothesise that:

*H1: COVID-19 perceptions will positively correlate with job insecurity.*

### COVID-19 and psychosocial issues

The relationships between job insecurity and psychosocial factors have been examined extensively. Such investigations include the meta-analysis work of [10, 30]. Much literature attempts to provide justification as to the linkages between job insecurity and adverse responses as far as psychosocial issues are concerned. Many researchers cite stress theory [31, 32] and the psychological contract violation [33]. The premise underlying stress theory is that job insecurity is a stressor that is difficult to cope with because it is not easy to control at the individual worker level. This leads to anxiety and psychological distress. Additionally, from a psychological contract theory perspective, workers exchange effort for pay and security of employment. A threat to job security is perceived as a violation of the latent psychological contract between the firm and the worker [33]. There is little question about the importance of hospitality workers given their direct association with customer satisfaction. Yet, workers assume arduous jobs with inflexible and long hours and have weak contract arrangements with limited union protections [34]. Hospitality work is almost by definition a precarious and fragile source of income [35], making it a precarious sector that affords workers little autonomy and low rewards [9]. Thus, the stress caused by work in the sector is further amplified during the COVID-19 pandemic, and we seek to understand the risk to hospitality workers health and well-being.

EB Faragher, M Cass and CL Cooper’s [36], which studied over 500 research papers, revealed the correlation between satisfaction with work and mental health conditions such as anxiety, depression and burnout. These correlations appear to be more pronounced than previously studied mental well-being aspects. In this perspective, job insecurity represents a work-life balance aspect that largely endangers the mental well-being of employees. We, therefore, anticipate that job insecurity can have clear links with negative psychosocial data [37]. These are significant parameters within the organisational commitment literature [38] and expectancy theory when workers contrast their old working conditions with current practices. We, therefore, hypothesise that:

*H2: COVID-19 perceptions positively predict negative psychosocial variables comprising anxiety, depression, burnout, and alienation.*

### Locus of control

We examine the possible moderating effect in our model based on individual differences by considering locus of control [15]. We reasoned that individuals might vary on how they experience the harmful effects of Covid-19 as a function of their own perceived control over situations. Individuals with internal locus of control will usually see themselves capable of managing externally-imposed events, using their own experience or self-efficacy. We then anticipate that model effects might be less pronounced among internals than individuals with external locus of control [39]. Scholarship into job insecurity (e.g. L Greenhalgh and Z Rosenblatt’s propositions) [40] stressed the moderating role of internal locus of control upon job insecurity.

Locus of control also moderates psychosocial reactions [41]. Hospitality employees’ job roles, moreover, afford them limited flexibility and autonomy due to the burden of supervision and menial nature of many of the tasks they are involved in. In this context, we suggest that internal locus of control may be associated with less pronounced adverse psychosocial reactions in comparison to external locus of control individuals. We, therefore, hypothesise that:

*H3: Locus of control moderates COVID-19 perceptions correlations with psychosocial factors.*

## Methods

The research focuses on the hospitality sector’s customer service workforce in the MENA region, covering Middle Eastern and North African countries. The data was collected via an online survey between April 2020 and March 2021. Participant recruitment was via the professional social network LinkedIn. The search criteria used were: country, job position, and hospitality area, e.g., restaurant, food & beverages, leisure, airline, travel & tourism, etc. As an online professional networking platform, LinkedIn enables job hunters to be resources for each other, sharing experiences and opportunities. The network is also a platform for recruiting companies that can source a variety of talents in a single online place [42]. LinkedIn was selected for participant recruitment owing to it being one of the world-leading professional networks, with more than 722 million users in over 200 countries [43]. The attempt to identify employees who work hospitality customer service in the MENA region yielded results in excess of 203,000. Our technique mirrors recent business psychology investigations that employed a similar sampling strategy [e.g., 44]. The researchers used a case in two counts after filtering the findings. The survey participants were advised about the research’s objectives and its methods and made aware of their right to withdraw from the study at any time if they wished to. The questionnaire had an area requiring their consent to participate in our survey. The participants’ responses were treated with anonymity and confidentiality. The five-minute questionnaire was made available in both Arabic and English. Our study returned 885 responses that were used in the analyses (response rate = 22%).

Scales formerly validated by AB Mahmoud, D Hack-Polay, L Fuxman and M Nicoletti [12] and AB Mahmoud, N Grigoriou, L Fuxman, WD Reisel, D Hack-Polay and I Mohr [45] in measuring COVID-19 perceptions, L Francis and J Barling [46] in measuring job insecurity, M Hamilton [47] in measuring anxiety, M Hamilton [48] in measuring depression, C Maslach and SE Jackson [49] in measuring burnout, D Lang [50] and M Banai and WD Reisel [51] in measuring alienation and PE Spector [52] measuring the work locus of control. A Likert scale based on 5 points was used to capture the responses provided by the participants. To assess the moderating role in the path model, we converted locus of control into a dummy variable which helped code as (0) people who exhibited an external locus of control and as (1) people who exhibited an internal locus of control.

The study used several indicators to ascertain the measures’ validity and reliability. The theoretical model was probed using the variance-based or Partial Least Square structural equation modelling approach (PLS-SEM) via SmartPLS 3 [53]. PLS-SEM received increased academic favourability when testing the predictive models [9, 54]. An important part of the data was predicted to depart from the criterion of multivariate normality [9]; thus, the PLS-SEM approach became apparent as a possible option for empirical probe in instances in which the data is sensitive to non-normality matters [55]. A path evaluation followed by multigroup analysis (MGA) was conducted to complement standardised betas (β: for direct effects), unstandardised betas (B: for indirect effects) and matching t-values through the adoption of bootstrapping, Q^2^ and PLSPredict for its predictive relevance and power and Cohen’s f^2^ to ascertain the effect sizes where f^2^ ≥ 0.02, f^2^ ≥ 0.15 and f^2^ ≥ 0.35 exemplify small, medium and significant effect sizes, respectively [56]. We also deployed the standard root mean square residual (SRMR) to assess the model fit to the data [57].

## Results

### Instrument validity and reliability

The Heterotrait-Monotrait Ratio of Correlations (HTMT) was computed and found to have values of less than .9, implying a satisfactory level of discriminant validity for all measures [58]. Furthermore, Table 1 indicates that all the constructs had average variance extracted (AVEs) higher than 0.5, composite reliability scores (CRs) between .78 and .90 satisfying the convergent validity and reliability criteria for all measures [51, 52]. Also, Table 2 shows that all Variance Inflation Factor values (VIFs) were less than 5, proving that collinearity is not a crucial issue [59].

**Table 1:** Discriminant validity test (HTMT)

	Alienation	Anxiety	Burnout	COVID-19 Perceptions	Depression
**Anxiety**	0.707				
**Burnout**	0.759	0.677			
**COVID-19 Perceptions**	0.253	0.361	0.259		
**Depression**	0.745	0.877	0.635	0.279	
**Job Insecurity**	0.736	0.569	0.548	0.38	0.501

**Table 2:** Outer loadings, VIFs, construct reliability and validity

	Alienation	Anxiety	Burnout	COVID-19 Perceptions	Depression	Job Insecurity	VIF
**ALIEN01**	0.677						1.838
**ALIEN02**	0.730						2.363
**ALIEN03**	0.738						2.277
**ALIEN04**	0.744						2.218
**ALIEN05**	0.727						1.311
**JSEC01**						0.738	1.545
**JSEC02**						0.791	1.780
**JSEC03**						0.723	2.082
**JSEC04**						0.631	2.041
**JSEC05**						0.698	1.914
**ANX01**		0.735					2.094
**ANX02**		0.698					2.034
**ANX03**		0.835					2.315
**ANX04**		0.817					1.765
**BURNOUT01**			0.773				1.556
**BURNOUT02**			0.766				1.893
**BURNOUT03**			0.680				1.606
**COV01**				0.688			1.297
**COV02**				0.748			1.574
**COV03**				0.814			1.383
**DEP01**					0.880		2.550
**DEP02**					0.880		3.174
**DEP03**					0.848		3.056
**Cronbach’s Alpha**	0.845	0.856	0.784	0.797	0.903	0.843	
**rho_A**	0.847	0.860	0.787	0.800	0.903	0.845	
**Composite Reliability**	0.846	0.856	0.784	0.795	0.903	0.841	
**Average Variance Extracted (AVE)**	0.523	0.598	0.549	0.566	0.756	0.516	

### Common method bias

Before examining the path and multigroup analyses, we ran Common-Method Bias (CMB) tests, which is considered a necessary procedure when using perceptual, self-report measures from a single survey [60]. The values of the inner variance inflation (VIFs) were all less than 3.3 (See Table 3). Consequently, we concluded that there were no multicollinearity or CMB issues found [61].

**Table 3:** Inner VIFs values

	Alienation	Anxiety	Burnout	COVID-19 Perceptions	Depression	Job Insecurity
Alienation				2.817		
Anxiety				2.564		
Burnout				1.763		
Depression				2.868		
Job Insecurity				1.595		
Alienation					2.46	
Anxiety					1.888	
Burnout					1.783	
COVID-19 Perceptions					1.14	
Job Insecurity					1.651	
Alienation		2.766				
Burnout		1.711				
COVID-19 Perceptions		1.126				
Depression		1.986				
Job Insecurity		1.649				
Alienation			2.426			
Anxiety			2.711			
COVID-19 Perceptions			1.142			
Depression			2.929			
Job Insecurity			1.669			
Alienation						2.245
Anxiety						2.583
Burnout						1.774
COVID-19 Perceptions						1.099
Depression						2.749
Anxiety	2.77					
Burnout	1.591					
COVID-19 Perceptions	1.138					
Depression	2.577					
Job Insecurity	1.358					

### Sample description

Using SPSS version 26, the majority of the sample were male (55%), millennials (45%) educated to a university degree (39%), single (51%), and with external locus of control (59%). Table 4 shows the descriptive statistics of the variables under investigation clustered into locus of control groups. It suggests that those with an external locus of control reported higher levels of COVID-19 perceptions, job insecurity, alienation, anxiety, depression, and burnout.

**Table 4:** Constructs’ descriptive statistics

Construct	Full sample (***N*** = 885)		Spilt based on locus of control levels			
			Internal (***N*** = 363)		External (***N*** = 522)	
	Mean	STDV	Mean	STDV	Mean	STDV
**Alienation**	3.148	1.231	2.695	1.099	3.463	1.220
**Job Insecurity**	3.102	1.140	2.797	1.067	3.315	1.142
**Anxiety**	3.527	1.136	2.990	1.197	3.901	0.923
**Burnout**	3.084	1.186	2.637	1.195	3.395	1.077
**COVID-19 Perceptions**	3.710	1.071	3.601	1.137	3.786	1.016
**Depression**	3.144	1.318	2.510	1.289	3.586	1.147

### Path analysis

Given the reflective nature of the latent variables in our model [9], we performed Consistent-PLS Algorithm, followed by Consistent PLS Bootstrapping run at 5000 sub-samples [62] in order to analyse the hypothesised path model.

COVID-19 perceptions are found to positively predict job insecurity (β = .363, *P* < .001, f^2^ > .15, *P* < .001), anxiety (β = .353, *P* < .001, f^2^ > .15, *P* < .001), alienation (β = .268, *P* < .001, f^2^ > .02, *P* < .01), depression (β = .285, *P* < .001, f^2^ > .02, *P* < .01) and burnout (β = .268, *P* < .001, f^2^ > .02, *P* < .01). Consequently, we judge H1 and H2 as fully supported (See Table 5).

**Table 5:** Hypotheses 1 & 2 testing

Hypothesis	Path	β	***t***	f^**2**^	Decision
H1	COVID-19 Perceptions - > Job Insecurity	0.363^**^	9.043^**^	> .15^**^	Supported
H2	COVID-19 Perceptions - > Alienation	0.268^**^	6.481^**^	> .02^*^	Supported
	COVID-19 Perceptions - > Anxiety	0.353^**^	8.502^**^	> .15^**^	
	COVID-19 Perceptions - > Burnout	0.268^**^	6.401^**^	> .02^*^	
	COVID-19 Perceptions - > Depression	0.285^**^	7.14^**^	> .02^*^	

* *P* < .01; ** P < .001

As SRMR was .034 < .08, we elected to suggest that our hypothetical model was an excellent fit for the data [63]. First, the Q^2^ values of all predictors were found to be higher than null, suggesting clear predictive relevance (See Table 6). Second, we compared the root mean squared error (RMSE) and the mean absolute error (MAE) values with the recommended naïve benchmark produced by the PLSpredict method [64] for each of the dependent constructs’ indicators. Since the PLS-SEM analysis (compared to the LM) yielded higher prediction errors in terms of RMSE (or MAE) for the minority number (see Table 7), we concluded that our model had a medium predictive power. Additionally, the *R*^2^ values of job insecurity (.132), anxiety (.125), depression (.082), burnout (.073) and alienation (.073) were higher than null, which meant that our formulated model had significant predictive validity, according to J Cohen [56].

**Table 6:** Predictive relevance (Q^2^)

	SSO	SSE	Q^**2**^ (=1-SSE/SSO)
Alienation	4370	4235.653	0.031
Anxiety	3496	3284.56	0.06
Burnout	2622	2543.724	0.03
Depression	2622	2495.317	0.048
Job Insecurity	4370	4121.001	0.057

**Table 7:** Predictive Performance of the PLS Model vs Benchmark LM

	PLS		LM	
	RMSE	MAE	RMSE	MAE
**ALIEN01**	1.632	1.472	1.624	1.452
**ALIEN02**	1.507	1.329	1.526	1.341
**ALIEN03**	1.626	1.482	1.638	1.491
**ALIEN04**	1.463	1.271	1.476	1.278
**ALIEN05**	1.56	1.392	1.579	1.402
**JSEC01**	1.335	1.151	1.332	1.138
**JSEC02**	1.297	1.104	1.296	1.087
**JSEC03**	1.318	1.09	1.314	1.076
**JSEC04**	1.469	1.245	1.475	1.251
**JSEC05**	1.45	1.254	1.466	1.266
**ANX01**	1.347	1.128	1.349	1.132
**ANX02**	1.438	1.238	1.451	1.243
**ANX03**	1.431	1.238	1.438	1.24
**ANX04**	1.476	1.262	1.485	1.282
**BURNOUT01**	1.443	1.248	1.456	1.253
**BURNOUT02**	1.414	1.197	1.409	1.188
**BURNOUT03**	1.444	1.251	1.462	1.26
**COV01**	1.473	1.302	1.443	1.252
**COV02**	1.558	1.385	1.572	1.403
**COV03**	1.31	1.091	1.31	1.102

### Multigroup analysis

We assess the path model invariance across the two groups of locus of control (i.e., internal vs external) through running a multigroup analysis (MGA). However, before that, we need to ensure that measurement invariance is established as a prerequisite to MGA [65].

J Henseler, R-JBJ R. Sinkovics, R Daekwan Kim, CM Ringle and M Sarstedt [65] contend that running group comparisons through PLS-SEM can be “misleading” unless the equivalency of their measures is confirmed [66]. By utilising the “Measurement Invariance of the Composite Models” (MICOM) technique, this condition may be fulfilled [65, 66]. Therefore, before conducting any multigroup investigations (in our particular instance: “with no data pooling”), both configural equivalency and compositional invariance need to be established and verified [59, 65–67]. Because we are using a PLS-SEM technique, we are able to achieve measurement configural invariance as a matter of course [59, 66]. Then, as a result of that, we ran a permutation test. The results are that all this study’s variates/constructs have their “Permutation *P*-values” more than 0.05; thus, we consider the null hypothesis to be accepted—meaning that the initial correlations of these constructs are non-substantially “different from 1” [66, 67] (See Table 8). This result offers valid evidence of compositional equivalency, implying a feasible multigroup assessment [59, 66].

**Table 8:** Compositional invariance assessment

	Original Correlation	Correlation Permutation Mean	5.00%	Permutation ***p***-Values
**Alienation**	0.979	0.899	0.587	0.778
**Anxiety**	0.962	0.982	0.954	0.067
**Burnout**	0.998	0.932	0.755	0.96
**COVID-19 Perceptions**	0.997	0.989	0.96	0.582
**Depression**	0.992	0.991	0.976	0.227
**Job Insecurity**	0.96	0.965	0.895	0.227

**Fig. 1:**
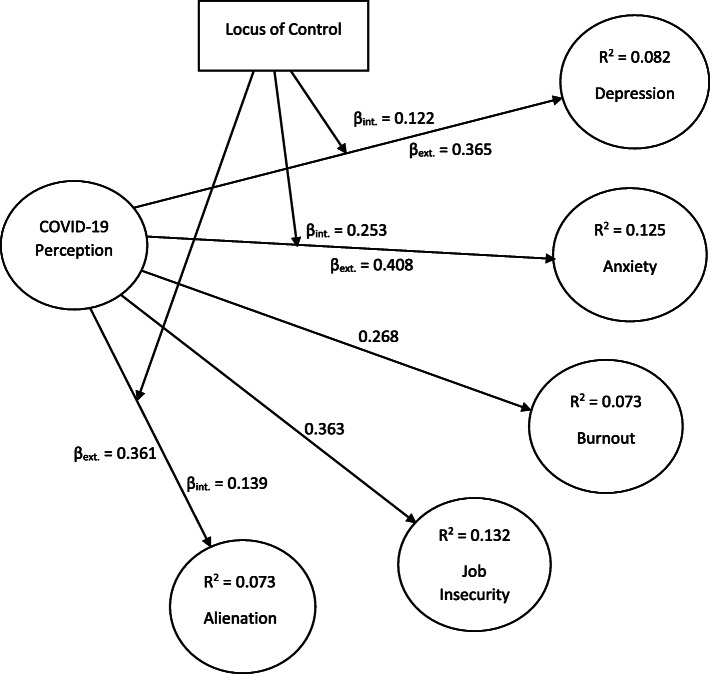
Hypotheses testing results

With regards to the MGA, the t-values linked to the multiple comparisons and shown in parametric tests are analysed. All the paths are significant for any of locus of control groups (significance level = .01). It was nonetheless the case that the paths: COVID-19 perceptions == > anxiety, COVID-19 perceptions == > depression and COVID-19 perceptions == > alienation are significantly non-equivalent between the two groups. From Fig. 1, the findings that customer service employees with external locus of control are more vulnerable to develop anxiety (β_int._ = .253 < β_ext._ = .408, *t*_ext. vs int._ = 2.756, *P* < .01), depression (β_int._ = .122 < β_ext._ = .365, *t*_ext. vs int._ = 3.973, *P* < .001) and alienation (β_int._ = .139 < β_ext._ = .361, *t*_ext. vs int._ = 3.342, *P* < .01) as consequence of their perceptions of COVID-19. Because all the paths are not moderated by the locus of control, it was concluded that H3 is supported only partially (See Table 9).

**Table 9:** Hypothesis 3 testing – Multigroup invariance analysis

Path	β_**int.**_	β_**ext.**_	***t***-Value (Internal vs External Locus of Control)
COVID-19 Perceptions - > Job Insecurity	0.337^**^	0.331^**^	0.140^*NS*^
COVID-19 Perceptions - > Alienation	0.139^*^	0.361^**^	3.342^*^
COVID-19 Perceptions - > Anxiety	0.253^**^	0.408^**^	2.756^*^
COVID-19 Perceptions - > Burnout	0.173^**^	0.232^**^	0.964^*NS*^
COVID-19 Perceptions - > Depression	0.122^*^	0.365^**^	3.973^**^

* *P* < .01; ** *P* < .001; *NS* = Non-significant

## Discussion

The research has investigated the effects of the coronavirus pandemic (COVID-19) perceptions within the hospitality sector in the MENA (Middle Eastern and North Africa) countries. We aimed to investigate whether COVID-19 perceptions predicted perceived job insecurity and negative psychosocial emotions and feelings amongst frontliners in the MENA hospitality sector. Further, we attempted to test whether those relationships could be moderated by locus of control as a personality trait. The central premise for our research propositions was that COVID-19 represents an exceptionally tough condition for the MENA hospitality sector workers, mainly frontliners, and is a significant impediment to employees’ well-being. Then, we used empirical data and theoretical frameworks (stress and work-life balance and psychological contract theories [31–33] as well as job insecurity issues prevalent in occupational health research [68] to model our hypotheses. The researchers’ initial perception was that generally, a crisis situation positively correlates with employees’ perceptions of job insecurity and individual effects [13–16, 37, 69, 70]. Our research is one of the first to examine these issues amidst the ongoing coronavirus crisis. Our focus is on the hospitality employees because they have limited options to avoid exposure since hospitality work is not convertible to virtual working and does not offer substitution from the physical work setting [19]. The research also seeks to establish how COVID-19 effects might be less pronounced among workers reporting an internal locus of control. This is about demonstrating how the perception of being in control of the situation helps manage the possible COVID-19 effects.

It is accepted that the workforce in the hospitality sector is critical to profit and firm viability [71]. Notwithstanding this vital role, employees in the sector find themselves excluded from generous employment terms and conditions. Instead, they face work overload, limited development opportunities, low pay and inflexible working patterns [72]. The sector’s workforce also must deal with the ever-present risk of exposure to the coronavirus as a function of the need to interact with customers directly. Our goal is to contribute to both theoretical and practical understanding with the hope that we might suggest possibilities to managers to limit the adverse effects given the prospect of worsened levels of organisational outcomes [e.g., 45]. This paper, therefore, adds a novel perspective for firms to respond to COVID-19 and protect the well-being, personal attitudes, and behaviours that are at the very centre of hospitality productivity.

Three key hypotheses were forwarded, building a model of COVID-19 effects on job insecurity and psychosocial reactions that we tested alongside the moderating effect of locus of control. We used partial-least-square structural equation modelling through which we ran path and multigroup analyses. The findings supported all three hypotheses. Hypothesis 3 addresses the moderating role of locus of control, which was less strongly supported. Our results suggested that the variances of job insecurity and burnout between internal and external types of people concerning locus of control are not as clear as the other constructs (i.e., anxiety, alienation and depression) of which participants with external locus of control reported more susceptibility as a response to COVID-19 than the internals. This implies that there are limits to individual dispositions in addressing potent and complex forces to cope with, such as COVID-19.

The findings are interesting and provide additional evidence about issues of job insecurity and psychosocial responses in the MENA region. The literature suggests that workers can see their employment as being volatile and unstable for several reasons, including the state of the economy or crises in the sector, organisational restructuring, quality of manager-employee relationships, resource availability and demographic factors [19]. Our evidence is among the first to associate a global health crisis with job insecurity accompanied by surges in adverse emotional reactions. We reason that the COVID-19 pandemic is detrimental to workers and organisational functioning. The study used an independent measure of COVID-19 perception [12] and found it to positively predict job insecurity, anxiety, depression, burnout and alienation. Hence, we contribute to the literature on job insecurity amidst crises, meaning that employees perceive health crises, e.g., COVID-19, as a threat to their jobs. This discovery aligns with evidence from prior research on COVID-19 negative impacts on employees in the services sector [45, 68]. Further, it concurs with previous research findings where COVID-19 impacts were not independently quantified and assessed [e.g., 3, 20]. Our research is consistent with the hospitality industry’s precarity reported in many previous studies [e.g., 34]. The negative work conditions cover various distressing aspects of hospitality work: chronic job insecurity, temporary contract status, and absence of union affiliation, which harm employees’ well-being. We further contribute to this line of evidence by adding the disruption of COVID-19 to the already adverse work conditions in the hospitality industry. As a consequence, prior research on poor well-being blames the incompetent human resources responses to the needs of hospitality workers [73]. Our data support this perspective and recommend that businesses should do more to demonstrate concerns about employee well-being and respond to them directly.

The research findings provide modest support concerning the role of locus of control as a moderating variable for relationships between job insecurity and its outcomes [e.g., 74]. That is an intriguing qualification within the present research originated from our moderation analysis (Hypothesis 3) comparing effects for hospitality industry workers with internal versus external locus of control. We discovered that the effects of COVID-19 are more acute for workers who see the world as externally controlled. Specifically, hospitality workers with a more positive sense of self-efficacy, ability to cope with and control over work conditions are less susceptible to the negative consequences of COVID-19 perceptions for the variables exhibited in our model. This result is in keeping with previously reported findings that highlighted externally controlled individuals as more vulnerable to stressful events than those internally controlled [75, 76].

### Practical implications

The major implications of the study in the MENA region show that hospitality workers who are in direct contact with customers have a higher perception of job insecurity, anxiety, depression, burnout and alienation as a result of COVID-19, and this will, as several empirical research investigations show, adversely impact the well-being, attitudes, and behaviours of employees. Our study has offered evidence suggesting positive relationships between COVID-19, job insecurity and psychosocial factors; this suggests that organisations should place emphasis on those initiatives that are within their control as COVID-19 is largely beyond the scope of a management team to address on a corporate level. However, as has been shown in prior research, managers are able to manage, to some degree, COVID-19’s adverse effects on hospitality workers through a statement about their compliance with statutory health guidance and establish the necessary supervisory arrangements for employees whose roles expose them more critically to COVID-19.

Moreover, as recommended by D Bangwal and P Tiwari [77], who considered environmental factors that influence job satisfaction and subsequent intention to quit, we too suggest an important role for management which should offer resources such as masks, vaccination, sanitiser, and other direct firm-level supports, including supervisory support, to address burdens introduced by the spread of COVID-19 on hospitality employees.

The effects of COVID-19 on employee mental well-being and job security are significant. It is, therefore, timely for organisations to develop plans to attempt to remedy the associated psychological distress. Another key finding is related to the moderating role of locus of control. We wanted to see if dispositions might alter the anticipated effects. We anticipated that workers with external locus of control would react poorly to the shock of the pandemic, and that is exactly what we learned. This implies that employees who begin with poorer self-efficacy and confidence in their capability to shape external events are going to respond poorly to public health crises such as COVID-19. External locus of control, therefore, raises significant challenges to management to enhance employee reactions to COVID-19 since locus of control is dispositional before attitudinal and thus hard to change. What we concluded is that COVID-19 is largely outside of any individual, business, or governmental entity’s control, so it is hard to suggest how management, independently, might shelter employees who view the world as externally controlled from the negative reactions to COVID-19.

### Research limitations and future perspectives

We acknowledge some limitations in our investigation. The first is linked to the broad nature of the sample; however, it is selective regionally as a result of the data being from the MENA region only. Authors such as H Kabasakal, A Dastmalchian, G Karacay and S Bayraktar [78] contend that the broad socio-cultural practises in the MENA context are thought to have high power distance scores implying high levels of expectations and acceptance of unequal power distribution in the region. The region also scored high on collectivism (i.e., the degree of individual expression of pride, cohesiveness and loyalty to their social groups). Future research could replicate this work in other regional contexts or undertake cross-cultural research using our model to establish the ways in which the results of our study might vary in different settings. Since our study did not record the participants’ country of work or residence, future research might consider this by replicating our study with the country of work or residence counted as a potential moderator. Secondly, our investigation was based on employees in the hospitality sector because they are more impacted by COVID-19 due to their in-person involvement in service provision during the pandemic than employees who can work from home. Such limitations show that more research is required to broaden the sample and job categories. Thirdly, although hospitality employees are likely to be underemployed, our choice of recruiting participants through LinkedIn might be associated with a bias towards educated people, especially with 39% of our sample were educated to a degree level. Fourthly, We needed to shorten the survey aiming for a better response rate [79] and minimized respondent fatigue [80] whilst bearing in mind the aim and objectives of this study; therefore, we purified the scales as to what items to eliminate was made based on the opinion of a panel of four research experts in the area of occupational health and psychology—a common practice in similar survey-based investigations in social science [10, 81]. Although the psychometric properties were sufficiently established for all the measures employed in this study, further research, however, might replicate our study with the whole items of each measure included in the survey.

Finally, a cross-sectional design was used for testing our proposed model, so it can be limited in terms of definitively establishing causal relationships between variables. Further research is required to capture longitudinal data to study in more depth the impacts and causal relationships. However, using a longitudinal design to reflect causality, as argued by PE Spector [82], has been exaggerated and that it only presents limited benefits over the cross-sectional design in most instances in which it is employed. Also, according to the findings of P Tharenou, R Donohue and B Cooper [83], research conducted using a cross-sectional investigation may not necessarily lack interpretability and validity provided they are supported by sound theoretical foundations. Cross-sectional research is also suitable for crises such as the coronavirus pandemic [e.g., 84]. What is needed for future research is to go further than just the use of locus of control as a variable. Future research could deploy more moderators encapsulating personality and demographics and work-life balance issues in the MENA context. This will aid managerial understanding and assist in operationalising the research findings.

## Conclusions

The main goal of the current study was to establish whether COVID-19 perceptions predict job insecurity, anxiety, burnout, alienation and depression amongst the MENA region’s hospitality frontliners. Moreover, this study set out to investigate if the effects of the COVID-19 perception might pronounce differently between externally and internally controlled hospitality frontliners in MENA to establish the moderating role of locus of control. The research employed a partial least squares structural equation modelling approach (PLS-SEM), drawing upon a sample of 885 participants to test the hypotheses by running path and multigroup analyses. With support for the hypotheses, this research originality centres on establishing that COVID-19 has a severe negative impact on the customer service labour force (in the MENA hospitality sector), triggering feelings of anxiety, burnout, alienation, and depression. Moreover, these effects were more profound for participants who claimed external locus of control than those with internal locus of control. Before this research, evidence of COVID-19 perceptions effects on psychosocial factors was anecdotal primarily due to not employing an independent measure to capture the personal experience of pandemic time.

## Data Availability

The data are not publicly available because they contain information that could compromise research participant privacy/consent.

## Abbreviations

COVID-19: Coronavirus pandemic
